# “The medications are the decision-makers…” Making reproductive and medication use decisions among female patients with rheumatoid arthritis: a constructivist grounded theory

**DOI:** 10.1186/s13075-021-02704-7

**Published:** 2022-01-22

**Authors:** Nevena Rebić, Sarah Munro, Ria Garg, Glen Hazlewood, Neda Amiri, Corisande Baldwin, Stephanie Ensworth, Laurie Proulx, Mary A. De Vera

**Affiliations:** 1 Faculty of Pharmaceutical Sciences, University of British Columbia, Vancouver, BC Canada; 2 Arthritis Research Centre of Canada, Vancouver, BC Canada; 3 Collaboration for Outcomes Research and Evaluation, Vancouver, BC Canada; 4 Centre for Health Evaluation and Outcome Sciences, Vancouver, BC Canada; 5 Department of Obstetrics and Gynaecology, University of British Columbia, Vancouver, BC Canada; 6 Cumming School of Medicine, University of Calgary, Calgary, AB Canada; 7 Faculty of Medicine, Department of Medicine, Division of Rheumatology, University of British Columbia, Vancouver, BC Canada; 8 Canadian Arthritis Patient Alliance, Ottawa, ON Canada

**Keywords:** Rheumatoid arthritis, Pregnancy, Medication use, Qualitative study

## Abstract

**Objective:**

To examine how female patients with RA form decisions about having children, pregnancy, and medication use.

**Methods:**

We employed a constructivist grounded theory design and recruited female participants who are 18 years or older, have a rheumatologist-confirmed RA diagnosis, live in Canada, and are able to communicate in English or French. We collected data through semi-structured individual and focus group interviews using telephone or video conferencing technology. Data collection and analysis were iterative, employed theoretical sampling, reflexive journaling, and peer debriefing, and culminated in a theoretical model.

**Results:**

We recruited 21 participants with a mean age of 34 years and median 10 years since RA diagnosis. Overall, 33% had never been pregnant, 57% had previously been pregnant, and 10% were pregnant at the time of interview. Of those who had experienced pregnancy, 64% had at least one pregnancy while diagnosed with RA and of those, 56% used DMARD(s) during a pregnancy. We constructed a patient-centred framework depicting the dynamic relationships between 4 decision-making processes—(1) using medications, (2) having children, (3) planning pregnancy, and (4) parenting—and the substantial impact of healthcare providers on patients’ experiences making these decisions. These processes were further influenced by participants’ intersecting identities and contextual factors, particularly attitudes towards health and medications, disease onset and severity, familial support system, and experiences interacting with the healthcare system.

**Conclusion:**

Our framework provides insight into how patients make reproductive decisions in the context of managing RA and the opportunities for providers to support them at each decision-making process. A patient-centred care approach is suggested to support female patients with RA in making reproductive and medication choices aligning with their individual desires, needs, and values.

## Introduction

Managing rheumatoid arthritis (RA) during pregnancy is a therapeutic challenge. Recent evidence suggests a decrease in RA activity in 60% of pregnant patients [1, 2] with only 20–40% attaining remission by the third trimester of pregnancy [3, 4]. Consequently, the majority of female patients with RA require some form of medication treatment during the perinatal period [5]. Nevertheless, several studies show low utilisation and considerable discontinuation of pregnancy compatible medications for RA perinatally [6–9]. Moreover, female patients with RA have information needs about medications in pregnancy [10–13], despite recent evidence-based guidelines supporting the safety of some disease-modifying anti-rheumatic drugs (DMARDs) in pregnancy [14–17].

Female patients with RA have fewer biological children than desired and as compared to other female individuals [18–20]. Reasons for smaller family size may include fertility issues, disease- and medication-related decrease in sexual activity, concerns about caring for a child (e.g., due to disease-related physical and functional limitations), uncertainty around medication use (e.g., stopping medications, adverse pregnancy and foetal outcomes), and fears about RA hereditability [19–25]. Despite recognition that females with RA are not meeting their reproductive goals, there are no studies examining the process of reproductive decision-making; specifically, the trade-offs related to managing disease activity and potential pregnancy. We aimed to develop a constructivist grounded theory of patients’ reproductive decision-making within the context of living with RA.

## Methods

### Design

Informing this ‘MOTHERS’ study is a Feminist Intersectional framework for understanding the relational and cumulative nature of independent systems of privilege and oppression that shape individual health and lead to health disparities [26–28]. Our approach was guided by constructivist grounded theory, which aims to generate theory to explain human phenomena and acknowledges the researcher and participants as co-constructers of its meaning [29–33]. Our analysis used both inductive and deductive approaches to conceptualise decision-making processes. Our patient research partner (LP) was involved throughout the research process, including grant submission, interview guide development, recruitment, data interpretation, and knowledge translation. This study was approved by the Behavioural Research Ethics Board at the University of British Columbia.

### Participants

We recruited participants using posters in rheumatology clinics across Canada and social media posts through investigators’ and patient organisations’ channels. Female participants were eligible if they were 18 years or older, had a rheumatologist-confirmed RA diagnosis, lived in Canada, and were able to communicate in English or French. Interested participants were provided a study URL to a questionnaire administered by the online survey platform Qualtrics through which they provided written informed consent electronically and responded to demographic information to aid in scheduling interview sessions and adapt the interview guide to their specific pregnancy and rheumatic disease experiences. We purposively sampled participants [34] to ensure diversity with respect to pregnancy intentions and experiences, disease and medication taking history, and province of residence. Moreover, we specifically sought participants who had never been pregnant, who had been pregnant and used medication, and who had been pregnant and not used medication.

### Data collection

We collected data through semi-structured synchronous video or telephone one-on-one interviews (~1 h) and online focus groups (~2.5 h) using the iTracks platform. We conducted focus groups with participants with similar pregnancy experiences (e.g., never been pregnant, previously pregnant and used RA medications) when possible. One bilingual author (NR) conducted the English interviews and was present to guide French interviews conducted by a Research Coordinator fluent in French. Interviews were digitally recorded, professionally transcribed, and translated to English where relevant. Data collection and analysis were simultaneous, with analysis informing subsequent data collection, and iterative, continuing until we achieved saturation of themes.

### Analysis

Our coding procedures included steps of line-by-line, focused, and theoretical coding. We used line-by-line coding to organise data into concepts and key phrases followed by focused coding to identify and group in vivo codes into categories [33]. Then, we used theoretical coding to identify connections and relationships between categories to provide insight into possible theories [33]. We employed constant comparison of data within and between transcripts to elevate the analysis to a conceptual level [33, 35]. Data analysis was conducted by the first author (NR) using NVivo 12. To support this process, NR created memos and conceptual diagrams to practice critical reflexivity, facilitate theoretical integration, and develop the resultant patient-centred grounded theory framework. To mitigate the impact of being an outsider without personal experience of living with a rheumatic disease or making healthcare decisions while considering the effects of those choices on a pregnancy, NR regularly consulted and debriefed with a patient partner, collaborator, and co-author (LP), an insider, throughout the research process.

## Results

We recruited 21 participants with a mean age of 34 years and median 10 years since diagnosis (Table 1). Most (86%) were married or co-habited with a romantic partner. All participants had a post-secondary education, most (67%) were employed full time, and the majority reported white (71%) or Asian (24%) ancestry.

**Table 1:** Participant characteristics

Characteristics	Statistic
Age (years), mean (range)	34 (21–46)
Years diagnosed with RA, median (IQR)	10 (2–16)
Language, *n* (%)	
English	20 (95)
French	1 (5)
**Canadian province of residence**, n (%)	
British Columbia	9 (43)
Ontario	7 (33)
Nova Scotia	2 (10)
Other (i.e. Alberta, New Brunswick, Québec)	3 (14)
**Geographic residence**^a^	
Rural	5 (24)
Urban	16 (76)
**Ancestry**, *n* (%)^b^	
White	15 (71)
Asian	5 (24)
Black	1 (5)
Hispanic	1 (5)
Indigenous	1 (5)
**Highest level of education**, n(%)	
Post-secondary (university, college, technical school, etc.)	21 (100)
**Current employment status**, n (%)^b^	
Employed full time (40 or more hours per week)	14 (67)
Student	4 (19)
Employed part time (less than 40 hours per week)	2 (10)
Unable to work	2 (10)
Unemployed and looking for work	1 (5)
**Household income** (CAD dollars), n (%)^c^	
150,000 and over	5 (25)
120,000 to 150,000	2 (10)
90,000 to 120,000	6 (30)
60,000 to 90,000	2 (10)
30,000 to 60,000	5 (25)
**Current members of household**, median (IQR)	3 (2 – 4)
**Marital status**, *n* (%)	
Married	13 (62)
Common-law or co-habiting	5 (24)
Single, never married	3 (14)
**Plan to have future children**, *n* (%)	12 (57)
**Future family planning options considered**, n (%)^b^	
Childbearing	12 (100)
Adoption	5 (42)
Other (i.e. surrogacy or assisted fertilisation)	4 (33)
**Medications taken for RA**, *n* (%)^b, d^	
**Traditional DMARDs**	
Antimalarials (i.e. hydroxychloroquine, chloroquine)	19 (91)
Methotrexate	15 (71)
Sulfasalazine	14 (67)
Leflunomide	4 (19)
Azathioprine	2 (10)
Gold salts	1 (5)
Cyclosporine	1 (5)
**Targeted DMARDs**	
Anti-tumour necrosis factor (anti-TNF) agents	13 (62)
Others (e.g. abatacept, rituximab, tocilizumab, tofacitinib)	7 (33)

^a^Rural is defined as less than 400 people per square kilometre. Urban is define as more than 400 people per square kilometre

^b^Cumulative percentage may be greater than 100, as multiple categories may be relevant to each participant

^c^Missing response from one participant

^d^Defined as any medication the participant has been exposed over the course of their RA treatment

Participants had diverse histories of RA, pregnancy, and medication use (see Table 2). Overall, 33% had never been pregnant, 57% had previously been pregnant, and 10% were pregnant during the interview. Of those who were pregnant or had previously experienced pregnancy, 64% had at least one pregnancy while diagnosed with RA and of those, 56% used DMARD(s), 33% used prednisone only, and 22% did not use medications during a pregnancy. Over half intended to have future children.

**Table 2:** Participant reproductive history and perinatal medication use descriptions

#	Pregnancy history	Medication use			Reproductive goals	Reproductive decision-making process^d^
		Preconception	Pregnancy	Post-partum		
P01	Never pregnant				To have children	Having children
P02	Never pregnant				To have children	Having children
P03	Never pregnant				To have children	Having children
P04	Never pregnant	Restarted MTX			To have children	Planning pregnancy, using medications
P05	Never pregnant	Using HCQ [↑ RA activity]			To have children	Planning pregnancy, using medications
P06	Never pregnant	Using chloroquine^c^			To have children	Pregnancy planning^c^
P07	Never pregnant				Finished growing family	
P08	No previous pregnancy, pregnant		Started HCQ^c^ and prednisone [↓ RA activity]		Intending to have children	Planning pregnancy^c^
P09	No previous pregnancy, pregnant	Stopped MTX, HCQ, SZS; started certolizumab pegol	Using certolizumab pegol [low RA activity]		To have children	Planning pregnancy
P10	Previous pregnancy^a^; 1 child	Stopped MTX; started certolizumab pegol	Used certolizumab pegol	Started MTX(?), stopped breastfeeding	To have children	Planning pregnancy
P11	Previous pregnancy^a^; 1 child	Stopped MTX, HCQ, SZS	Used prednisone; [high RA activity]	Started certolizumab pegol, delayed MTX to breastfeed	Finished growing family	
P12	Previous pregnancy^a^; 1 child	Used MTX and etanercept	Stopped MTX in T1; used etanercept, prednisone [high RA activity]	Started MTX, stopped breastfeeding	Finished growing family	
P13	Previous pregnancy^a^; 2^b^ children	Stopped SZS	Used prednisone [high RA activity]	Started MTX, stopped breastfeeding	Finished growing family	
P14	Previous pregnancies^a^, 2 children	1st: used etanercept until conception 2nd: Used etanercept	1st: no medication; [high RA activity] 2nd: Used etanercept [low RA activity]	1^st^: used etanercept; did not breastfeed 2^nd^: used etanercept; did not breastfeed	Finished growing family	
P15	Previous pregnancies^a^; 2 children	1st: ^c^Did not start DMARDs 2nd: Stopped HCQ	1st: no medication; [low RA activity] 2nd: no medication; [high RA activity]	1st: breastfeeding? [High RA activity] 2nd: Started tDMARDs; breastfeeding?	Finished growing family	
P16	Previous pregnancies^a^; 2 children	1st: Stopped LEF, MTX 2nd: Used prednisone	1st: Used prednisone 2nd: Used prednisone	2nd: Started DMARDs after breastfeeding	Finished growing family	
P17	Previous pregnancies; no children	^c^Using SZS, HCQ; intending to start bDMARD			Intending to have children	Having children
P18	Previous pregnancies; 4 children			Delayed MTX^c^ to breastfeed	To have children	Having children
P19	Previous pregnancy; 4 children	Using HCQ, etanercept			To have children	Planning pregnancy
P20	Previous pregnancy; 3^b^ children			Using HCQ^c^, SZS; delaying MTX to breastfeed	Finished growing family	Parenting
P21	Previous pregnancy; 1 child	^c^			Finished growing family	Parenting

Abbreviations: *bDMARDs* biologic DMARDs, *DMARDs* disease-modifying anti-rheumatic drugs, *tDMARDs* targeted DMARDs, *HCQ* hydrochloroquine, *LEF* leflunomide, *MTX* methotrexate, *RA* rheumatoid arthritis, *SZS* sulfasalazine

^a^Diagnosed with RA during one or more previous pregnancy experiences

^b^One child was not carried by participant through pregnancy (i.e., adopted or joined family through marriage)

^c^Indicates timing of receiving RA diagnosis if within the perinatal period

^d^Relative to the timing of the interview with participant

### Decision-making processes

**Fig. 1:**
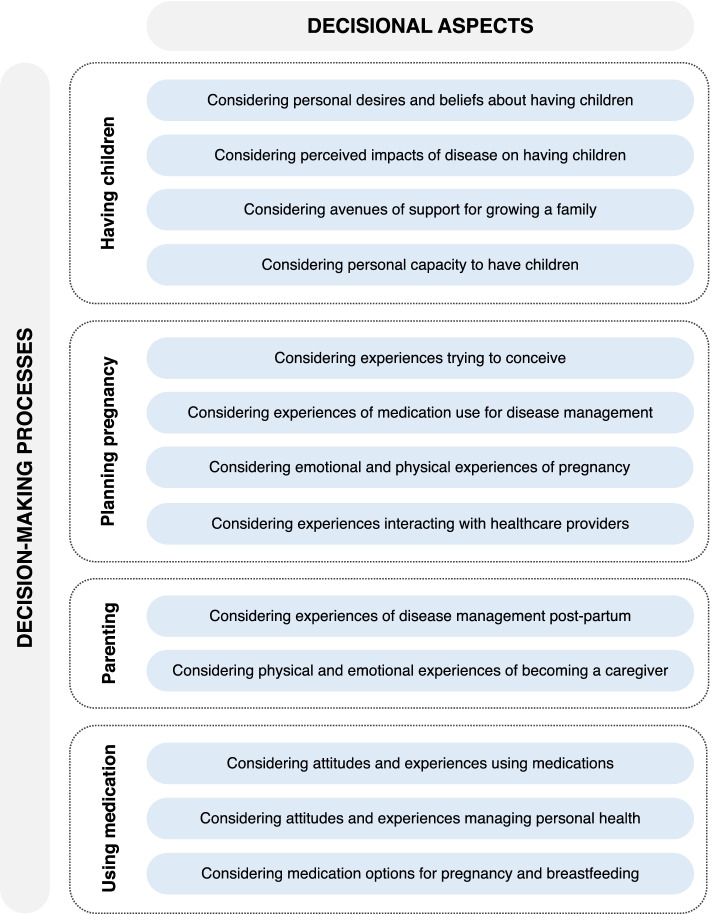
Decisional aspects of reproductive and medication use decision-making processes

We identified three dynamic, closely connected, and interactive decision-making processes related to participants’ reproductive decisions and experiences: (1) *having children*, (2) *planning pregnancy*, and (3) *parenting*. A fourth process, *using medications*, was present throughout participants’ reproductive lives, influencing the aforementioned decision-making processes. Contextual factors influencing these processes are presented in Fig. 1, with representative quotes presented in Table 3.

**Table 3:** Themes and representative quotations

**Decision-making process**	**Decisional aspects**	**Representative Quotations **
Having children	Considering personal desires and beliefs about having children	‘Once I finally came to the decision of not having kids then it was almost freeing in a way. I didn’t have to be like, “Is this something I want to do? Is this something I can do?” and then kind of sitting down with myself and realizing that “no, this isn’t what I want”.’ (Participant 7)
		‘We were always on the fence about having kids, so we had kids older. Then we loved that idea and we wanted our child to have a sibling so that’s why we were trying for a second. But even before I got diagnosed, age was always on my mind. And then [after] the diagnosis [we] pretty much just said, “Yeah, no, let’s not push it”.’ (Participant 21)
	Considering personal impacts of disease on having children	‘The medications are pretty much the decision maker. They rule this decision. Even if me as an individual decide, “Okay I want to get pregnant”, it’s waiting: “Well, how long does this medication, how long do you need to be off it so you can actually conceive?” And then, “How long can I be on another dose while in the gestation process, so it doesn’t affect the baby?” [My medication] rules all my decisions that I make before and throughout that whole process.’ (Participant 2)
		‘I have a really great mom and I always worried that if I had a child that that child wouldn’t get the same experience because maybe I would have too severe of arthritis and I wouldn’t be able to play with them or things like that […] always in the back of my mind was, “It wouldn’t be fair to them because I have such a good mom, I want [my child] to have one too”.’ (Participant 10)
	Considering avenues of support for growing a family	‘Another thing that factors in is the fact that [my partner] has a chronic disease and my mom, the grandmother [who has RA] would also have one […] Usually, let’s say me and [my partner] are really struggling, we could have grandparents come [help out] but [my mom’s] health is really not good right now and that makes me think.’ (Participant 1)
		‘[Having children is] not just a conversation between you and your partner, it’s a conversation between you, your partner, and your relevant healthcare providers.’ (Participant 11)
	Considering personal capacity to have children	‘I started talking to my husband about having kids very early on. [On] the first date I told him, “I want kids. If you don’t, there’s no point here.” He’s a couple years younger than me so the clock was ticking so we started talking about it very early on: “Do we want kids? How many do we want?” And I said, “We gotta start having them soon ’cause I’m a little bit older than you,” and I explained to him all the stuff with my RA and medications and how it went.’ (Participant 14)
		‘I got the big “absolutely not, you should not, even if you think there’s a possibility that you could get pregnant do not be on this medication” or at least that’s the fearmongering I got from my [rheumatologist]. [My rheumatologist] made the assumption that I’m done with my family and so this is the medication you have to take […] and it just felt like [they] were forcing this [medication] on me, like this choice that I wasn’t making for myself, that [they were] assuming my family plans are done and so it’s not a problem, just take this medication and that’s it, right? […] I’m fortunate in that I already have children but if I hadn’t and, “Wait a minute, am I even done having kids? Why is that decision being taken from me?”.’ (Participant 19)
Planning pregnancy	Considering experiences trying to conceive	‘[Methotrexate] was one of the [medications] I dropped right away because if it needs to be out of my system for six months or whatever the timeframe was, I wanted an extra few months because I don’t wanna take the risk […] That [medication] still made me a little bit nervous.’ (Participant 16)
		‘I remember when I went off of methotrexate and my husband [asked], “So how long do we keep trying if it doesn’t happen quickly?” There’s a limit to how much pain I can handle, even just mentally, but month to month when it impacts not just your day-to-day life, your work, how to get to and from work and household tasks. So that was definitely a part of [trying to conceive] and just being in constant pain without having full access to the [treatment] tools you could usually access when you aren’t pregnant or trying to become pregnant.’ (Participant 11)
	Considering experiences of medication use	‘When I found out I was pregnant, I went to see a walk-in doctor and that doctor panicked, “What? You’re on methotrexate? If it was up to me, I would give you the pill so you can have an abortion.” What?! So, that was probably one of the worst most frightening weekends I ever had in my life […] and this family doctor said stop all medications so with all the stress I didn’t take any medications on that weekend. And then on the Monday, I went back to the rheumatologist and [they] said, “Calm down, I’m not an expert but I don’t think you have to get rid of your child, not yet,” and [they] referred me to a specialist. But then for that specialist, they say you have to be 12 weeks pregnant to see [them] […] a five week wait, until I got to see the specialist was a complete nightmare. And my arthritis flared up horribly. […] I had no true management for those five weeks while I waited for this other doctor. Everybody was scared of saying anything to me.’ (Participant 12)
		‘[My rheumatologist] was very clear. “[…] Having chronic inflammation in your body, that’s not good for your growing baby either so you need to do something right now that we know is relatively safe for you and the baby and is gonna help you function,” and I’m so glad that I have taken the medications ’cause it’s made my quality of life so much better and it’s given me an opportunity to enjoy this pregnancy because between morning sickness and then joint pain, I hadn’t had a chance to do that at all.’ (Participant 8)
	Considering emotional and physical experiences of pregnancy	‘[During both pregnancies] I worried the whole entire pregnancy. So, the first pregnancy, I wasn’t worried so much about my baby other than regular worries that you have. I was more, “Am I gonna get my arthritis under control? Is my medication gonna work again [after pregnancy]? If it doesn’t, then I’m gonna have to go on [another] medication, what if that doesn’t work? What if I can’t pick my baby up?” All of those worries. And then my second pregnancy, it was just constant worry about what the medication is doing to the baby and guilt, constant guilt, “Should I have done this? Was this selfish just because I wanted another baby?” So, it definitely impacted my mental health with stress and anxiety […] I still have daily guilt, daily worry, and daily stress.’ (Participant 14)
		‘I remember when we were trying to conceive in particular, all of my medical decisions were being made based on what was best for a person that didn’t even exist yet and so there was six months where I was switching medication and then there was the almost two years of trying and then there was the nine months of pregnancy and then there was the breastfeeding and I got really, really fed up with it ’cause I just didn’t feel like I was my own person anymore. I was just the host for the baby so that kind of played on my mental health a lot.’ (Participant 10)
	Considering experiences interacting with healthcare providers	‘[With an RA diagnosis] it is automatically a risky pregnancy, so it’s immediately in the hospital. I hate hospitals, I hate residents who look at you as a case number and not like a person. I am someone who is hypersensitive to everything already therefore the fact of giving birth in the hospital, it stresses me out. In addition, the thought of giving birth it stresses me more, because of the facts of giving birth [as] someone with rheumatoid arthritis. I want to be like, “[…] can my baby just like materialize out of my body?”’ (Participant 6)
		‘I remember being quite annoyed about it actually, that it was like I stopped existing because I was pregnant… [My rheumatologist] was basically like, “Well, this medication that you’re on is not working so stop it and then since you’re gonna try and get pregnant, let’s not bother with anything until after ’cause your next step is a biologic and you can’t do that and be pregnant. So, that’s the only kind of treatment that they offer is: you’re on this medication or you’re not. And if you’re not on a medication, then, “Why would we see you?” So they said, “You just revert to the care of your [general practitioner, GP], don’t call us, we’ll call you.” […] After that, when I was in pain, I was phoning my GP and she was like, “Oh, it’s your round ligaments,” and I’m like, “It’s not my frickin’ round ligaments, it’s my joints!” and she was like, “Sorry, [your rheumatologist] won’t see you while you’re pregnant.’ (Participant 15)
Parenting	Considering experiences of disease management post-partum	‘[When] my two youngest were tiny, breastfeeding was also difficult because it involves your hands a lot and my hands and elbows are my most strongly affected and so even that was difficult in the early parenting days where I was having to hold a baby for hours a day […] I would do nursing side lying or just reclined so I tried to do as much of that as I could just to save my hands. I also used a lot of topicals, a lot of cold packs, NSAIDs, Voltaren [diclofenac] became my best friend. Obviously, you have to be a bit careful with the baby ’cause you don’t want him getting all numb but a lot of that and then just kind of sucking it up and coping with it.’ (Participant 18)
	Considering physical and emotional experiences of becoming a caregiver	‘It makes me emotional. […] I felt like a bad mother. I couldn’t pick her up. My husband always carried her. He did the toilet training. I couldn’t even lift her to put her on the toilet. She still goes to daddy for toilet ’cause that’s what she’s used to. Yeah, it was tough. Again, I still have fatigue issues, so breakfast time is daddy. […] Kids understand when mom is sick, so I think it’s affected her. She’s always very concerned when I’m not well, if I sneeze or anything, she’s, “Mommy are you okay?” She does artwork for me, so it’s definitely affected our way of living our life.’ (Participant 21)
		‘I honestly don’t know if I could handle being in a two-income household. Having my husband home is a huge help ’cause I don’t have to do a lot of cleaning. That is one thing that is a bit challenging ’cause of my wrists so the less repetitive strain that I have to put on my body to scrub shower stalls and stuff is a really big help. […]And having a bit of financial and childcare support from my family, it’s huge. It enables the lifestyle that we have ’cause if we had a two-way family household with daycare, it would be different. I would not be so functional.’ (Participant 20)
Using medications	Considering attitudes and experiences using medications	‘I'm someone who doesn’t like to take even [ibuprofen] or [acetaminophen]. I know that there is a place and a time for that but I’m not someone that runs to my medicine cabinet all the time. So, it was kind of a curve to come to the acceptance that I did need it [medications to manage my RA].’ (Participant 9)
		‘I struggled a long time to find [a medication that works]. I was on one medication and it was fantastic and then it stopped working after about eight years. And then there was about a year and a half of trying other things and trying to get myself settled again and that was really, really tough. I went from being able to walk and behave fairly undetected, most people didn’t even know I had RA, to everybody asking me what was wrong with me. It was huge. So, the idea of then stopping [my medication], that was terrifying. I didn’t know what I would be without it. […] I know some people can come off their medication and feel okay. I don’t think that I'm one of those people.’ (Participant 7)
	Considering attitudes and experiences managing personal health	‘It does make me wish that there was more of a holistic approach but it’s not really readily available or supported here. […] To say, just blanket, “Everything that is not medication [is not effective]” – no. And that’s [my rheumatologist’s] approach. [They are] completely and entirely drug focused. […] There’s not really any, “Here are your resources or here are supports that you can use to help you.” None of that is offered. And I think that's a damn shame.’ (Participant 15)
		‘I wanna be in good shape as much as possible to be able to take care of my kids and do things, so I constantly worry more now about, “If I take this, what could be the side effects for me in a couple years?” And, “If I do this, is this a smart thing or a good thing to do?” And it’s just made me a lot more anxious. In a way it’s good because I pay a lot more attention to that stuff now and I’ve learned a lot more about my body, but then I also found that, in terms of mental health, it’s just made me a lot more anxious in general.’ (Participant 13)
	Considering medication options for pregnancy and breastfeeding	‘I'm only on sulfasalazine and hydroxychloroquine now but probably moving to a biologic very soon but obviously trying to be in the realm where they’re all pregnancy safe type medications first. And obviously if my symptoms increase or they’re not receptive to what I’m taking then I’ll cross that bridge when I get there but that’s the mindset and the approach that I’ve been taking now because why would I start on medication that I would have to just stop or reduce or do something for my symptoms to recur when there are options out there that I could take?’ (Participant 17)
		‘It’s challenging to just dive on drugs or to change them up because there’s not that many options that you can use when you’re trying to get pregnant. My options are even more limited [because I’ve changed my therapy several times]. I don’t even know how many more [medications] there are for me to try. But it’s challenging to have to rely on something working to make your life livable.’ (Participant 4)
		*‘*It’s just a fear of, “What could happen?” […] It's the unknown, in terms of, “Could this impact a successful pregnancy? Could this cause cognitive issues or some sort of issues in my child down the road by taking such a strong medication?” Those are the things that I fear. […] To me [targeted DMARDs] haven’t been out that long so what I think of [is a scenario of] a woman who’s pregnant now and her 20-year-old has some sort of medical issue down the road and it’s because [she] took a biologic.’ (Participant 5)

### Having children

Participants described 4 decisional aspects influencing their decisions to prevent unplanned pregnancy and to choose whether to have children.

First, participants *considered their individual desires and beliefs about having children*, which were influenced by their age, expectations of motherhood, and mindset towards their childrearing options. Some ‘*always knew [they] wanted kids’* while others felt less certain about their reproductive desires. Attitudes towards the environmental impact of biological children, perceived and experienced physical impacts of pregnancy, societal messaging about being a woman and having children, and size and composition of their childhood family influenced how participants evaluated their options (e.g., childbearing, adoption, and fostering). RA further complicated this consideration. Some participants contemplated how adoption would lessen the impact of disease management but remove the ‘*intimacy’* of experiencing pregnancy. Some believed pregnancy was not an option due to needing to prioritize managing their RA in medication decisions, having insufficient perinatal support from their healthcare team, or receiving discouraging messages about pregnancy from healthcare providers. Regardless of outcome, having the option to become pregnant was important.

Participants also *considered the impact of their disease on having children*, with their understanding dependent on the timing of disease onset and disease severity. For some, RA ‘*forced*’ them to consider their reproductive desires earlier than their peers, including whether having children was ‘*feasible*’. While RA was not the deciding factor, participants considered it a ‘*complication*’ and additional ‘*barrier*’ to factor into their decision. Overwhelmingly, participants worried about the impact of pregnancy on their ability to manage RA perinatally. For some, ‘*just not knowing how pregnancy is gonna affect [their] RA*’, whether it would flare or go into remission, was reason to consider not experiencing pregnancy. While some participants perceived pregnancy compatible medications as an opportunity to consider having biological children, others worried having their drug therapy restricted to medications compatible with pregnancy limited their disease management options. Some also feared stopping medications that ‘*changed [their] life for the better*’ for pregnancy. Several participants wondered whether they ‘*could*’ become pregnant, sharing RA-related challenges with fertility and engaging in sexual activity. Most worried about being ‘*a good mom*’ and how their RA may ‘*unfairly*’ affect a child’s life, including fears about being able to ‘*raise a kid*’ and potentially ‘*passing on*’ their RA.

Moreover, participants *considered their avenues of support for growing a family*. Rheumatologists were influential in participants’ decisions to become pregnant and use medications perinatally. Participants with positive provider relationships felt supported, informed, comfortable discussing their reproductive options, and confident taking medications. Others struggled to have ‘*open conversations*’ about their reproductive goals with rheumatologists. Several participants believed female providers were more ‘*sensitive*’ to their reproductive care needs. Participants also considered their partner’s age, health, desire to have children, and capacity for fulfilling additional caregiving responsibilities. Two participants without a partner at the time of their decision, acknowledged that this impacted their decisions to delay pregnancy and not have a child. Overwhelmingly, participants’ partners actively supported them through learning about RA, engaging in discussions about reproductive planning, and listening and validating their worries. Additionally, participants considered the availability of childcare assistance from extended family.

Lastly, participants *considered their capacity to have children* within the context of their life, particularly their personal readiness and reproductive window for becoming pregnant. They envisioned achieving specific personal and professional milestones (e.g., completing their education, securing a permanent job, buying a house, being in a committed relationship) prior to having children. Additionally, the ‘*timing’* of their diagnosis, disease severity, and access to parental leave and insurance coverage for pregnancy compatible medications affected their decisions. Most participants actively prevented unplanned pregnancy, which was a source of anxiety and fear. Participants on teratogenic medications were vigilant in using ‘*consistent, reliable’* contraceptives. Two participants considering permanent contraception options (i.e., tubal ligation, vasectomy) once they finished having children. Although some participants considered age a limiting factor for timing pregnancy, older participants and those with children wanted to ‘*[keep] some agency*’ and not be discounted from having children.

### Planning pregnancy

Participants who were planning pregnancy, were pregnant, or had previously experienced pregnancy described 4 decisional aspects influencing their conception and pregnancy decisions.

Participants *considered their experiences trying to conceive*, with their desired pregnancy timelines often at odds with timeframes for medication changes and conception with RA. Medications were the ‘*first thing*’ diagnosed participants considered when planning and timing pregnancy. For some, ensuring safe pregnancy removed the ‘*romance*’ and ‘*spontaneity*’ of trying to conceive with their partner. Often, conception required more time than expected, with some experiencing disease flares during this period. Additionally, some participants felt pressured to conceive quickly by their rheumatologist. Three participants were referred to a fertility specialist and one took medications to aid with conception.

Participants *considered their experiences of medication use for disease management* pre-conception and during pregnancy. Participants’ perinatal medication choices are reported in Table 2. Prior to planned conception, participants followed medication recommendations from their rheumatologists, with some stopping DMARDs compatible with pregnancy and others starting biologic DMARDs (bDMARDs). Five participants used DMARDs during pregnancy. Some felt reassured their medications were safe while others worried about potential risks. Participant 12 experienced an unplanned pregnancy while using a teratogenic medication. Overall, most participants recognised the importance of controlling their disease activity for their own and their baby’s health.

Participants who had experienced pregnancy *considered their emotional and physical experiences of pregnancy*, reporting mixed experiences with disease activity (see Table 2). All three participants who only used prednisone experienced flares during pregnancy, while two of the three participants who used bDMARDs maintained good disease control. Participant 12 experienced active disease while on a bDMARD. Of the two participants who did not take any medications, one went into remission during their first pregnancy but experienced active disease during their second pregnancy, while the other experienced a flare during their first pregnancy and decided to use DMARDs during their second pregnancy. Two participants diagnosed postnatally suspected they experienced untreated RA during pregnancy. All diagnosed participants who became pregnant reported feeling anxious throughout pregnancy planning about ‘*things that could wrong*’ related to the uncertainty of perinatal disease management. Many shared needing to ‘*let go of any expectations*’ and navigate ‘*one thing at a time*’. Those using medications shared concerns about perinatal medication use, with some deciding to maintain a positive outlook and ‘*trust in the science*’. When making medication decisions, many participants noted ‘*focusing on the baby’*, which at times came at a detriment to their own health and disease management.

Finally, participants *considered their experiences interacting with healthcare providers*. Those with planned pregnancies worked closely with rheumatologists to make medication and pregnancy decisions. Participants using medications were closely monitored by their healthcare team throughout the perinatal period. Although this was reassuring to most, for some it caused additional anxiety. Notably, two participants who stopped all medications preconception felt unsupported by their healthcare team as they were not closely monitored.

### Parenting

Participants with an RA diagnosis during pregnancy or post-partum described 2 decisional aspects influencing their parenting decisions.

Participants *considered their experiences of disease management post-partum*. Six reported experiencing post-partum flares, which influenced their infant feeding decisions (i.e., whether to breastfeed and for how long). Of those who breastfed, some delayed starting medications incompatible with breastfeeding and two used compatible bDMARDs. Another two stopped breastfeeding to start new medications. Some participants described physical challenges of breastfeeding, particularly if RA affected their arms or wrists. Most participants considered breastfeeding an important motherhood experience and opportunity for ‘*connection*’ with their baby. Several shared the harm of encountering dogmatic messages such as ‘*breast is best*’.

Relatedly, participants *considered their emotional and physical experiences becoming a caregiver* to a child, with many feeling ‘*unprepared’* to navigate the impacts of RA on caring for a baby. While most felt relieved post-partum, some shared anxieties about their decisions, including questioning whether they could have ‘*done something different*’ and ‘*prevented*’ unwanted outcomes (e.g., premature delivery). Some worried about long-term impacts of perinatal medication use on their child, even if ‘*the research looks great*’, and changing their medications for pregnancy putting their health on a ‘*different track five or ten years down the road*’. Many participants experienced physical challenges taking care of newborns which required ‘*strategic planning*’ (e.g., related to infant dressing, picking up children, and navigating disease-related fatigue). Participants described prioritising being a ‘*present*’ parent by purposefully allocating their limited energy and identifying RA-friendly family activities. Overall, parenting with RA involved accepting that their children would have different experiences than their peers and recognising that growing up more independently taught their children resilience, empathy, and responsibility. All participants with children shared a caregiver role with a romantic partner and described how they divided parenting duties, including having their partners ‘*take charge*’ of more physically involved tasks. Some further received help from family and friends, including assistance with carrying their child, childcare, and financial support. Feeling limited in their capacity to perform tasks attributed to motherhood took an emotional toll on participants.

### Using medications

Throughout their reproductive years, participants described 3 decisional aspects influencing their decisions to start, use, and stop DMARDs and analgesic medications.

Participants *considered their attitudes and experiences using medications* broadly. Although some were not accustomed to taking medications regularly prior to diagnosis, all came to realise they needed medications to function. This required time and a grieving process before reaching acceptance. Some depicted not having a choice if they wanted a ‘*livable*’ life. Several participants described struggling to find effective medications at some point in their treatment. For some, it was important to minimise medication exposure by taking ‘*as little medications as possible*’ and the ‘*mildest*’ medication, which was often related to concerns about long-term medication use. Participants discussed the importance of being presented with choices in their care, particularly with medications that affect their reproductive options.

Participants also *considered their attitudes and experiences managing their health*. Overall, participants perceived their RA management was medication focused. Although participants recognised the importance of medications, they wanted rheumatologists to support other aspects of disease management, including non-medication guidance and referrals for their physical, nutritional, mental, and sexual health. Those with children discussed how motherhood motivated them to ‘*[pay] more attention to [their] body*’, including recognising when they needed to prioritize their health to ‘*be a strong mamma’*.

Finally, participants *considered their medication options for pregnancy and breastfeeding*. Some wanted to prioritize starting ‘*pregnancy safe medications first’* to avoid a potential flare when stopping incompatible medications preconception. Most participants, particularly those who had tried several different therapies, felt their pregnancy options were limited. Although newer bDMARDs provided additional treatment options, some feared unknown long-term effects on an exposed child’s health that may be identified in the future. Participants acknowledged that their perception of the safety of medication use was strongly influenced by their rheumatologist’s beliefs and attitudes towards medications.

### Patient-centred constructivist grounded theory

**Fig. 2:**
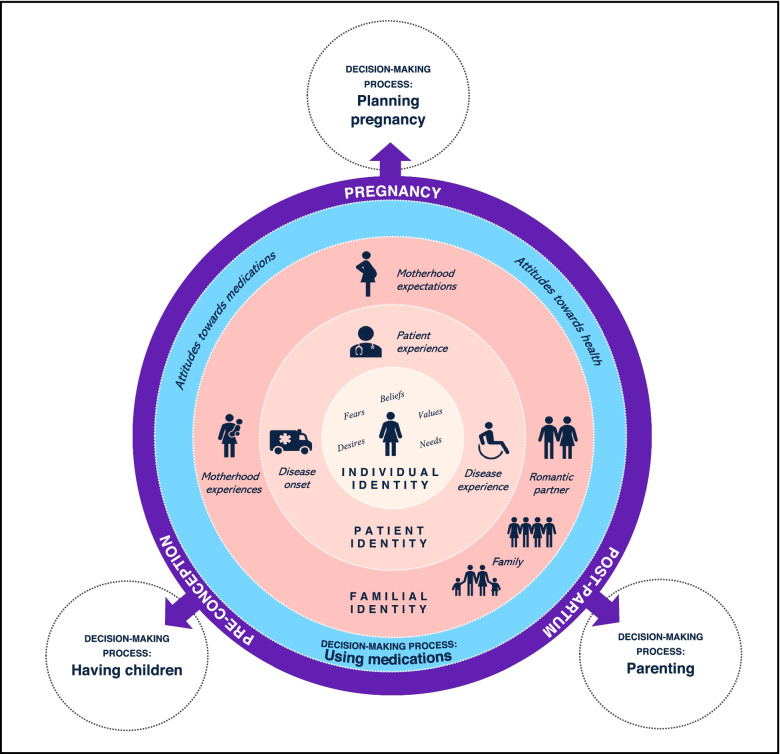
A constructivist, patient-centred, grounded theory of reproductive and medication use decision-making while living with RA

Our resultant theory (shown in Fig. 2) centres the female patient among contextual factors that form their decision-making environment as they navigate reproductive decision-making processes while living with RA.

The patient’s reproductive window is set within the black box encompassing the figure. Encircling the patient are contextual factors related to being a woman (*individual identity*), a person with a chronic disease (*patient identity*), and a member of a family (*familial identity*) that influence their reproductive and medication use decision-making environment. These identities are fluid, overlapping, and influence one another. The emotional effect of interactions between these identities is depicted by the rose-coloured gradient, which is overwhelmingly dominated by fluctuating acute and chronic experiences of anxiety, fear, and guilt – emotional experiences resulting from the degree of confidence the patient experiences within the decision-making processes and their capacity to cognitively and emotionally address their psychological state. These identities and contextual factors are related to their *reproductive journey* (purple circle) pre-conception, during pregnancy, and post-partum and associated temporally with the reproductive decision-making processes: (1) *having children*, (2) *planning pregnancy*, and (3) *parenting*. The fourth decision-making process, (4) *using medications*, occurs in a dynamic context between an individual’s identities, reproductive journey, and reproductive decision-making processes.

## Discussion

Our study is the first to examine reproductive and medication decision-making among female patients with RA. Our patient-centred constructivist grounded theory demonstrates the fundamental role of medication use and disease management in patients’ reproductive decision-making—‘*The medications are pretty much the decision maker’* (Participant 2). Moreover, our results reveal that contextual factors—individual desires, fears, beliefs, values, and needs, disease and patient experiences, expectations and experiences of motherhood, and support from partners, family, and friends—influence how patients form decisions about having children, planning pregnancy, and using medication. Our theory’s dynamic and multidimensional structure reflects the complex and relational nature of women’s lives with implications for supporting a patient-centred approach to delivering reproductive care within rheumatology practice.

Our results have implications for improving perinatal standards of care for patients with RA. Despite research showing female patients with RA stop their medications during pregnancy [6–9] and have information needs regarding perinatal medication use [10–13], no studies have examined how they approach these decisions. Given the results from our study examining this phenomenon, we recommend that rheumatologists initiate discussions with patients about their current and future pregnancy prevention (e.g., contraception) and support (e.g., medication management, interdisciplinary referrals) needs early in the therapeutic relationship. Addressing reproductive topics pre-emptively and regularly ensures rheumatologists are supporting patients in preventing unplanned pregnancies on teratogenic medications and establishing a plan for managing RA perinatally. Building patient trust may be particularly important among male rheumatologists, with whom female patients may face additional barriers discussing reproductive care. We further recommend that rheumatologists continue to see patients regularly throughout pregnancy, including those who stop medications as they are more likely to experience pregnancy flares with an associated increased risk of untoward pregnancy outcomes. Post-delivery, rheumatologists are well situated to support breastfeeding decisions and provide referrals to RA-friendly resources for parenting. Finally, given the risks of unintended pregnancy post-partum [36], rheumatologists can support patients starting teratogenic medications or seeking to prevent subsequent pregnancy establish a contraception plan.

Our results reflect others [37–39] that show patients’ perception of their provider relationship and healthcare experiences are key aspects of being activated in their care and that well-informed patients feel more confident and empowered in healthcare decision-making. Collaborations with rheumatologists and patient partners strengthened our data collection, analysis, and interpretation. Remote interviews facilitated recruiting a geographically diverse sample; however, recruitment was limited to individuals with access to telephone and video conferencing technology. Our data collection was further enriched through facilitating focus groups among participants with similar pregnancy histories. It is important to note that our sample comprised predominantly white and highly educated participants in heterosexual relationships. Further studies should examine the applicability of our theory to patients with historically marginalised racial and sexual identities as well as diverse family structures (e.g., single parent families). As systemic barriers to healthcare access contribute to significant gaps in care, higher disease activity, and poorer pregnancy outcomes among Indigenous, Black, and Hispanic patients with rheumatic diseases [40–43], our findings suggest an imminent need to address reproductive and medication decision-making needs among racialized patients.

## Conclusions

Our study identified how gaps in care impact patients’ reproductive decisions and perceived capacity to grow their family. By understanding the practical and emotional aspects of patients’ decision-making processes, healthcare providers can identify opportunities for intervention and care adaptation leading to better reproductive choices that align with patients’ individual desires, values, and needs.

## Data Availability

The data used in this article cannot be shared publicly due to institutional ethics restrictions intending to protect the privacy of research participants.

## Abbreviations

bDMARD(s): Biologic DMARD(s)
DMARD(s): Disease-modifying antirheumatic drug(s)
RA: Rheumatoid arthritis
URL: Uniform resource locator
